# Correction: Leppäniemi et al. Nutrition Profile for Countries of the Eastern Mediterranean Region with Different Income Levels: An Analytical Review. *Children* 2023, *10*, 236

**DOI:** 10.3390/children11081005

**Published:** 2024-08-16

**Authors:** Hanna Leppäniemi, Eman T. Ibrahim, Marwa M. S. Abbass, Elaine Borghi, Monica C. Flores-Urrutia, Elisa Dominguez Muriel, Giovanna Gatica-Domínguez, Richard Kumapley, Asmus Hammerich, Ayoub Al-Jawaldeh

**Affiliations:** 1 Regional Office for the Eastern Mediterranean (EMRO), World Health Organization (WHO), Cairo 7608, Egypt; leppaniemih@who.int (H.L.); eibrahim@who.int (E.T.I.); hammericha@who.int (A.H.); aljawaldeha@who.int (A.A.-J.); 2 Oral Biology Department, Faculty of Dentistry, Cairo University, Cairo 11553, Egypt; 3 Department of Nutrition and Food Safety, World Health Organization, 1211 Geneva, Switzerland; borghie@who.int (E.B.); floresm@who.int (M.C.F.-U.); domingueze@who.int (E.D.M.); gaticag@who.int (G.G.-D.); kumapleyr@who.int (R.K.)

In the original publication [1], there was a mistake in Figure 1, showing the countries of the Eastern Mediterranean Region, as published. The border of Morocco has not been shown correctly on the map. The corrected Figure 1 (which correctly represents the borders of Morocco) appears below. The authors state that the scientific conclusions are unaffected. This correction was approved by the Academic Editor. The original publication has also been updated.

## Figures and Tables

**Figure 1:**
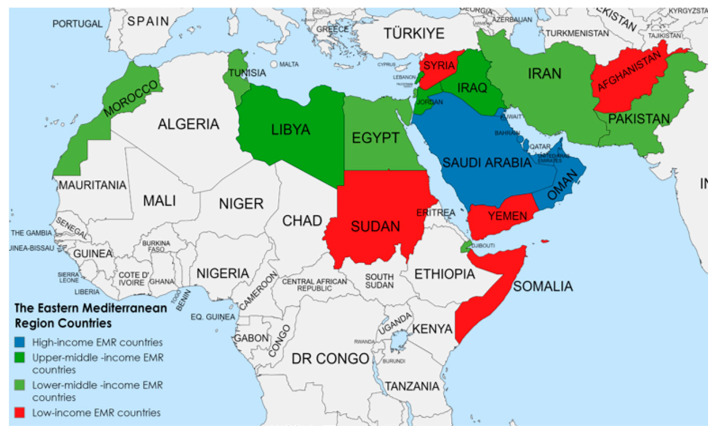
The countries of the Eastern Mediterranean Region.
